# Long-term outcomes of early initiated antiretroviral therapy in sub-Saharan children: a Cameroonian cohort study (ANRS-12140 Pediacam study, 2008–2013, Cameroon)

**DOI:** 10.1186/s12887-021-02664-6

**Published:** 2021-04-21

**Authors:** Francis Ateba Ndongo, Mathurin Cyrille Tejiokem, Calixte Ida Penda, Suzie Tetang Ndiang, Jean-Audrey Ndongo, Georgette Guemkam, Casimir Ledoux Sofeu, Paul Alain Tagnouokam-ngoupo, Anfumbom Kfutwah, Philippe Msellati, Albert Faye, Josiane Warszawski

**Affiliations:** 1 Université Paris-Sud, Centre Mère et Enfant de la Fondation Chantal Biya, Francis, POB 1936, Yaounde, Cameroon; 2 Centre Pasteur du Cameroun, Service d’Epidémiologie et de Santé Publique, Yaounde, Cameroon; 3 MPH, PH-PU, Université Douala; Hôpital Laquintinie, Douala, Cameroon; 4 Centre Hospitalier d’Essos, Yaounde, Cameroon; 5 Centre Mère et Enfant de la Fondation Chantal Biya, Yaounde, Cameroon; 6 Université Yaoundé I; Centre Pasteur du Cameroun, Service d’Epidémiologie et de Santé Publique, Yaounde, Cameroon; 7 Centre Pasteur du Cameroun, Service de Virologie, Yaounde, Cameroon; 8 TransVIHMI, IRD, Montpellier, France; 9 Université Paris Diderot, Sorbonne Paris Cité; Assistance Publique des Hôpitaux de Paris, Pédiatrie Générale, Hôpital Robert Debré, INSERM UMR 1123, ECEVE, Paris, France; 10 Université Paris-Sud, Assistance Publique des Hôpitaux de Paris, CESP INSERM U1018, team 4 “HIV and STD”, Hôpital Bicêtre, 94276 Le Kremlin-Bicêtre, France

**Keywords:** HIV-infected children, Early initiated antiretroviral treatment, Long-term outcomes

## Abstract

**Background:**

In most studies, the virological response is assessed during the first two years of antiretroviral treatment initiated in HIV-infected infants. However, early initiation of antiretroviral therapy exposes infants to very long-lasting treatment. Moreover, maintaining viral suppression in children is difficult. We aimed to assess the virologic response and mortality in HIV-infected children after five years of early initiated antiretroviral treatment (ART) and identify factors associated with virologic success in Cameroon.

**Methods:**

In the ANRS-12140 Pediacam cohort study, 2008–2013, Cameroon, we included all the 149 children who were still alive after two years of early ART. Virologic response was assessed after 5 years of treatment. The probability of maintaining virologic success between two and five years of ART was estimated using Kaplan-Meier curve. The immune status and mortality were also studied at five years after ART initiation. Factors associated with a viral load < 400 copies/mL in children still alive at five years of ART were studied using logistic regressions.

**Results:**

The viral load after five years of early ART was suppressed in 66.8% (60.1–73.5) of the 144 children still alive and in care. Among the children with viral suppression after two years of ART, the probability of maintaining viral suppression after five years of ART was 64.0% (54.0–74.0). The only factor associated with viral suppression after five years of ART was achievement of confirmed virological success within the first two years of ART (OR = 2.7 (1.1–6.8); *p* = 0.033).

**Conclusions:**

The probability of maintaining viral suppression between two and five years of early initiated ART which was quite low highlights the difficulty of parents to administer drugs daily to their children in sub-Saharan Africa. It also stressed the importance of initial viral suppression for achieving and maintaining virologic success in the long-term. Further studies should focus on identifying strategies that would enhance better retention in care and improved adherence to treatment within the first two years of ART early initiated in Sub-Saharan HIV-infected children.

## Background

Over the last years, the beneficial effects of antiretroviral therapy (ART) have been reported among HIV-infected children [1–4]. Early initiation of ART in infants and young children has been proven to be associated with reduced morbidity and mortality [5–13]. Based on such evidence, ART treatment guidelines of the World Health Organization (WHO) and various countries have been updated to recommend early initiation of ART in HIV-infected infants [14–17], leading to an increase in child enrolment in sub-Saharan African pediatric ART programs [18–20].

In most studies, the virologic response is assessed during the first two years following ART initiation of HIV-infected infants [7–10, 12, 21–28]. Early initiation of ART in vertically HIV-infected children exposes them to a very long-lasting treatment relative to that of adults. Maintaining viral suppression in children is very difficult [8, 29, 30]. Pediatric ART options are limited in Sub-Saharan Africa due to low availability of pediatric formulations [16, 22, 31, 32]. Thus long-term success of early infant ART in Sub-Saharan Africa depends on the efficacy of first-line regimens [33, 34]. There is a need to study longer-term efficacy of early initiated ART in HIV-infected children.

In the ANRS-12140 Pediacam cohort study, Cameroonian HIV-infected children were initiated on ART at a median age of four months [35, 36]. We aimed to determine the virologic response and mortality after five years of antiretroviral treatment initiated during the first year of life based on data from this cohort study and identify factors associated with virologic success in a sub-Saharan country (Cameroon).

## Methods

### The ANRS-12140 Pediacam study

ANRS-12140 Pediacam is a prospective cohort study of HIV-infected children included between November 2007 and October 2011 from three referral hospitals in Cameroon: the “Centre Mère et Enfant de la Fondation Chantal Biya” (CME-FCB) and “Centre Hospitalier d’Essos” (CHE), both in Yaounde, and the “Hôpital Laquintinie de Douala” (HLD) in Douala. Pediacam comprised two phases and has been described previously [35, 37]. Briefly, during the first phase, infants born to HIV-infected mothers and those born to HIV-uninfected mothers were matched according to gender and site of recruitment during the first week of life and followed until the fourteenth week. HIV-exposed infants were tested for HIV using Polymerase Chain Reaction (PCR) test at six weeks of age, according to the Cameroonian guidelines at the time of the study. Results of HIV test were available at 10 weeks with retesting for HIV-positive diagnoses. HIV-negative breastfed infants were retested six weeks after weaning. All HIV-infected infants and subsamples of uninfected HIV-exposed and HIV-unexposed infants were eligible for the second phase of follow-up, planned to continue until five years of age. During the above inclusion period, direct inclusion into the phase 2 follow-up was proposed to the mothers of HIV-infected infants identified and diagnosed after the first week but before seven months of life. Overall, 210 HIV-infected infants were included in this second phase. Antitretroviral therapy (ART) was systematically proposed as soon as the HIV status was confirmed, according to the Cameroonian guidelines at the time of the study: zidovudine (or abacavir or stavudine) + lamivudine, for all infants, combined with ritonavir-boosted lopinavir or nevirapine, depending on the previous history of nevirapine used for prevention of mother-to-child transmission (PMTCT). Viral load of HIV was measured by RT PCR (Biocentric, Bandol, France) using plasma specimen, with 300 copies/mL as lower limit of quantification from 2007 to 2011. Thereafter, new devices for measuring viral load were acquired with the threshold of 60 copies/mL as lower limit of quantification. Data concerning biological and clinical parameters were collected at inclusion and every three months after ART initiation until the age of two years and every six months thereafter. At the same time-points, a standardized questionnaire was administered to caregivers to collect data on family living conditions and adherence to ART. Children whose HIV viral load remained high (≥1000 copies/mL) for at least three consecutive medical visits were selected for HIV genotypic resistance test to ARVs performed with 1 mL of plasma using the French ANRS (French National Agency for Research on AIDS and Viral Hepatitis) protocol [38]. The targeted regions were Protease and Reverse transcriptase of the HIV polymerase gene. The exams and transport fees were paid by the Pediacam project.

### Study population

**Fig. 1:**
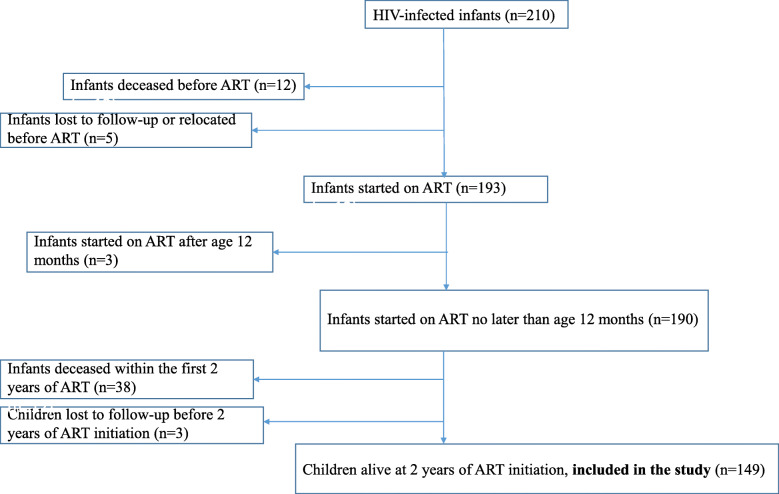
Flow chart of HIV-infected infants initiating ART (ANRS-Pediacam Study, 2008–2013, Cameroon). **ART:** Antiretroviral therapy

We considered for this analysis the HIV-infected children included in phase 2 of the Pediacam study who were alive at two years of ART initiated no later than the age of one year (Fig. 1). Data were analyzed from the second to fifth year of ART.

### Endpoints

The main outcome variable for this study was virologic success (VS), defined on the one hand as the achievement of viral load < 400 copies/mL at least once during the period from two to five years after ART initiation, and one the other as the achievement of viral load < 400 copies/mL after five years of ART initiation. This threshold had also been reported in previous studies [9, 24, 39–41]. We also studied mortality and the immune status, defined by the percentage of CD4 lymphocytes, at five years after ART initiation.

Virologic, immunological, and clinical data were analyzed during the visits scheduled every six months from 24 months after ART initiation: month 24 (M24), month 30 (M30), month 36 (M36), month 42 (M42), month 48 (M48), month 54 (M54), and month 60 (M60). A window of three months before and after these time points was accepted for data collection. The monitoring values used were the nearest measurements within these ±3-month intervals. The children still alive who did not attend the scheduled visit were classified as virologic failure with respect to the corresponding visit.

### Exposure variables

We mainly considered characteristics at M24 to identify factors associated with VS at M60: calendar period, ART regimen, site of ART delivery, age and clinical presentation of the child, attainment of confirmed VS (at least two consecutive viral loads (VL) < 1000 copies/mL) achievement of confirmed virological success (at least two consecutive viral loads < 1000 copies/mL) at least once within the first two years after ART initiation) [42], virological status at M24 (viral load < or ≥ 1000 copies/mL), and immunological status (percentage of CD4 lymphocytes). We also considered variables related to living conditions at M24: people living in the household, access to running water, access to electricity, and availability of a functional refrigerator at home. Finally, we considered the reporting of caregiver adherence (recall of missed doses in the past three days) collected at M60. Children were classified as non-adherent if at least one dose was missed during the past three days [18, 36].

### Statistical analysis

The study population was initially divided into two groups by distinguishing their virological status at two years of ART: 1) children who had a viral load < 400 copies/mL at M24 (Group 1) and 2) children who had a viral load ≥400 copies/mL or whose viral load had not been measured at M24 (15 children of whom the last viral load measured was ≥400 copies/mL at M12) (Group 2). The characteristics at two years of treatment according to a viral load below or above 400 copies/mL were compared by grouping together the children whose viral load was ≥400 copies/mL or not measured.

The probability of maintaining VS from M24 to M60 for group 1 was estimated using the Kaplan-Meier model. The probability of achieving VS for group 2 was estimated using a competing risk regression curve (with death as the competing event).

The association of exposure variables with viral load < 400 copies/mL (versus a viral load ≥400 copies/mL) at M60 for children who were still alive was assessed in univariate and multivariate analysis using logistic regression. The site of ART delivery, virological status at M24, ART regimen at M24, and reporting of caregiver adherence (recall of missed doses in the past three days) at M60 were included in all multivariate models (a priori risk factors). Other exposure variables, with *p* ≤ 0.20 in univariate analysis, were also included in the initial multivariate model. The final model was built using backwards elimination, comprising a priori risk factors and other exposure variables with *p* ≤ 0.20. All statistical analyses were performed using STATA© 13.0 (Stata Corp, College Station, TX).

## Results

### Study population

Among the 210 HIV-infected children included in phase 2 of the Pediacam study, 149 (71.1%) who initiated ART no later than the age of one year were alive at two years and considered for this analysis (Fig. 1). Of the 61 HIV-infected children who were not included in this study, 12 (19.7%) where deceased before ART, 5 (8.2%) were lost to follow-up or relocated before ART, 3 (4.9%) were placed on ART after age 12 months, 38 (62.3%) deceased within the first two years of ART and 3 (4.9%) were lost to follow-up before 2 years of ART initiation. Among the 149 children (82 boys) followed-up and still alive at two years after ART initiation, with ART started during the first year of life, almost three quarters (73.9%) were enrolled in the two Yaounde sites (Table 1). The median age of the children at two years after ART initiation (origin of time for this analysis) was 28.5 months (IQR: 27.1–30.0). Most (75.6%) were living with their mothers (with or without their fathers) in households of which 39.4% had running water, 93.6% electricity, and 51.1% a functional refrigerator. The ART regimen two years after treatment initiation was comprised of lopinavir boosted with ritonavir (74.5%) or nevirapine (25.5%), with a median percentage of CD4 lymphocytes of 33.6% (24.1–40.4).

**Table 1:** Baseline characteristics of HIV-infected children alive at 24 months after early antiretroviral treatment initiation (ANRS-Pediacam Study, 2008–2013, Cameroon) (*N* = 149)

	N	% (n) or median (IQR)
**Male**	149	55.0 (82)
**Site of ART delivery**	149	
CME/FCB		51.7 (77)
HLD		22.2 (33)
CHE		26.1 (39)
**At 24 months after ART initiation (Study start)**		
**Age at 2 years after ART initiation**	149	
< 28 months (treatment initiated before age 4 months of age)		50.3 (75)
[28–30[months (treatment initiated between age 4 and 6 months)		30.2 (45)
≥ 30 months (treatment initiated from 6 months of age)		19.5 (29)
*Median in months (****IQR****)*		28.5 (27.1–30.0)
**Time period**	149	
2010–2011		65.1 (97)
2012–2013		34.9 (52)
**ART regimen**	149	
Lopinavir-based		74.5 (111)
Nevirapine-based		25.5 (38)
**Children living with**	131	
Both parents		38.9 (51)
Mother only		36.7 (48)
Father only		5.3 (7)
Other relatives		19.1 (25)
**Family size ≤ 5 people**	94	47.9 (45)
**Access at child’s home to**	94	
Refrigerator		51.1 (48)
Running water		39.4 (37)
Electricity		93.6 (88)
**CD4 percentage at 2 years after ART initiation**	131	
< 15		6.1 (8)
[15–20[		6.1 (8)
20–25[		14.5 (19)
≥ 25		73.3 (96)
*Not measured*		*13*
*Median (****IQR****)*, *N = 131*		33.6 (24.5–41.6)
**Virological status**		
**Achievement of confirmed virological success (CVS) within the first two years of ART**	149	
Yes (=2 VL < 1000 copies/mL for two consecutive measurements)		77.2 (115)
No		22.8 (34)
**Virological status at 2 years (±3 months) after ART initiation**	134	
VL < 400 copies/mL		67.2 (90)
VL ≥ 400 copies/mL		32.8 (44)
*VL not measured*		*15*
**Last viral load if not available at 2 years ± 3 months after ART initiation**	15	
VL < 400 copies/mL		12
VL ≥ 400 copies/mL		3

*N*: total number of subjects; *n*: number of subjects in the category; *ART* antiretroviral therapy; *CME/FCB* Centre Mère et Enfant de la Fondation Chantal Biya; *HLD* Hôpital Laquintinie de Douala; *CHE* Centre Hospitalier d’Essos; *IQR* interquartile range; *VL* viral load

At two years after ART initiation, most of the children (77.2%) had achieved a confirmed VS at least once [42] and a viral load measurement was available for 134, of whom 67.2% were < 400 copies/mL («controlled viral load»). Among the 15 other children, the last available viral load was ≥400 copies/mL for 12 (80.0%).

The children with VS at two years of treatment were more often girls than boys (62.2% versus 37.8%, *p* = 0.017) and almost all (96.7%) had achieved confirmed virological success (CVS) at least once before two years of treatment, whereas this was true for only 45.5% of the other children (*p* < 0.001).

### Mortality and retention in care between two and five years after ART initiation

Among the 149 children alive at two years of treatment and included in this study, a viral load measurement was available at M60 for 121 (81.2%) and 23 (15.4%) did not have viral load measurements at five years of ART whilst they were still alive (Table 2). Of these, 12 (8.1%) did not attend any clinical visit since the time period before M24. Five (3.4%) deaths were recorded M24 and M60, corresponding to a death rate of 3.0% (95%CI: 0.2–5.8%) estimated using the Kaplan Meier method. Among them, 2 (40.0%) occurred in children with a viral load ≥400 copies/mL and 3 (60.0%) in children whose viral load was unavailable at two years of treatment, whereas there were no deaths among children with VS at two years of treatment.

**Table 2:** Mortality and immuno-virological status at 5 years (+/−3 months) of children with antiretroviral treatment initiated within the first year of life (ANRS-Pediacam Study, 2008–2013, Cameroon) (N = 149)

			Viral load at 2 years (+/− 3 months) of antiretroviral treatment initiation					
	All N = 149		< 4 00 copies/mL ***N*** = 90		**≥** 400 copies/mL ***N*** = 44		Absence of viral load ***N*** = 15	
	N	%	N	%	N	%	N	%
**Death between 2 and 5 years of ART initiation**								
Yes	5	3.4	0	0.0	2	8.5	3	20.0
No	144	94.6	90	100.0	42	91.5	12	80.0
**Virological status at 5 years (+/−3 months) after ART initiation**								
VL < 400 copies/mL	99	66.4	68	75.6	28	63.6	3	20.0
VL ≥ 400 copies/mL	22	14.8	9	10.0	12	27.3	1	6.7
Absence of VL if not dead at M60	23	15.4	13	14.4	2	4.6	8	53.3
**Probability at 5 years (+/−3 months) after ART initiation, using Kaplan-Meier estimation method**								
Of maintaining virological success (Group 1)	90	64.0 (30 events)						
Of achieving at least once VL < 400 copies/mL (Group 2)	44	76.0 (30 events)						
**CD4 percentage at 5 years of ART initiation**								
< 15	1	0.7	0	0.0	1	2.3	0	0.0
[15–20[	0	0.0	0	0.0	0	0.0	0	0.0
[20–25[	7	4.7	5	5.6	1	2.3	1	6.7
≥ 25	115	77.2	74	82.2	38	63.6	3	20.0
Not measured if not dead at M60	21	14.1	11	12.2	2	4.6	8	53.3

*N*: total number of subjects; *n*: number of subjects in the category; *ART* antiretroviral therapy; *IQR* interquartile range; *VL* viral load; *M60* 60 months of ART initiation; 95% CI: 95% confidence interval

### Immunological status at five years of antiretroviral treatment

Among the children included in this study, almost three quarters (77.2%) had a high CD4 percentage level (≥25%) at 5 years of ART (Table 2).

### Virologic control at five years of antiretroviral treatment

**Fig. 2:**
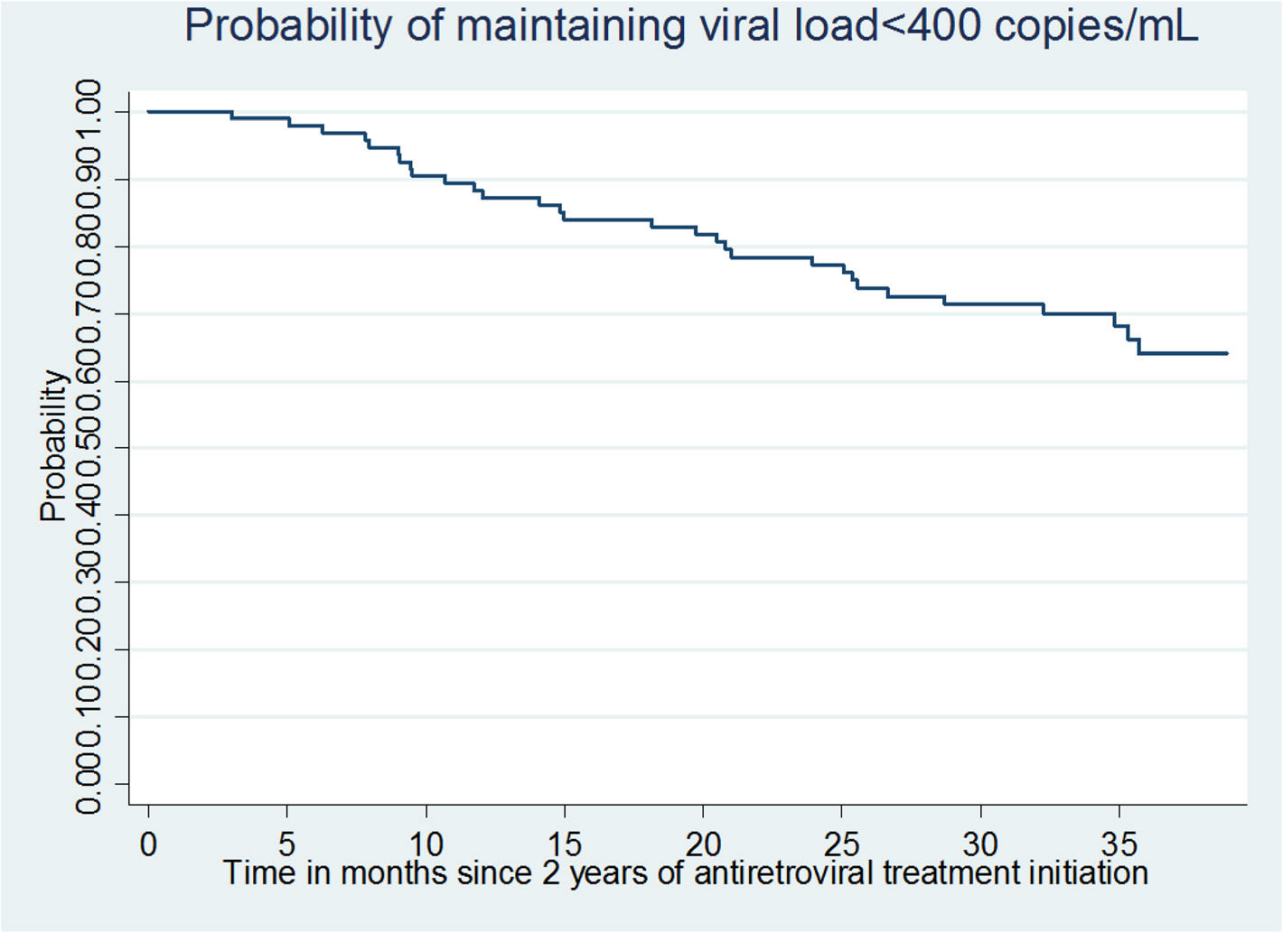
Kaplan-Meier curve: probability of maintaining a viral load < 400 copies/mL between 2 and 5 years after antiretroviral treatment initiation among the 90 children with a viral load < 400 copies/mL at 2 years after antiretroviral treatment initiation (ANRS-Pediacam Study, 2008–2013, Cameroon)

Virologic control at five years of ART was estimated at 66.8% (60.1–73.5) among the 144 children still alive after five years of treatment. The viral load measurement was not available in 23 (16.0%) of them at that deadline (Table 2). Among the children who were virologically controlled (viral load < 400 copies/mL) at two years of treatment, the probability of maintaining virologic control at five years, estimated using the Kaplan-Meier method, was 64.0% (54.0–74.0), (Table 2 and Fig. 2). Of those whose viral load was uncontrolled or unknown at two years of ART, the probability of achieving a viral load < 400 copies/mL between two and five years was 76.0% (63.0–89.0).

Genotypic resistance test of HIV to antiretroviral drugs was performed for a sample of 25 children whose viral load remained high (≥1000 copies/mL) for at least three consecutive medical visits during the study. Of these, 15 (60.0%) were boys and 18 (72.0%) have initiated lopinavir-based ART. The distribution of sex and type of ART in this sample was comparable to the study population. Among these children, 5 (20.0%) rebounded to viral load ≥1000 copies/mL during the study period for at least 3 consecutive visits before suppressing to viral load < 400 copies/mL no later than five years of ART. Overall, respectively 14 (56.0%), 9 (36.0%) and 2 (8.0%) of these children had no resistance, only lamivudine (3TC) resistance (not altering the effectiveness of current ART regimen) and resistance to non-3TC drugs (altering the effectiveness of current ART regimen). The resistance pattern did not differ by whether the child was suppressed or not at 5 years of ART, according to the Fisher exact test (*p* = 0.440).

### Factors associated with virological success at five years of antiretroviral treatment

In univariate analysis (Table 3), VS at five years of treatment (viral load < 400 copies/mL versus viral load ≥400 copies/mL or not measured) was associated with female gender (OR = 2.1 (1.0–4.4), *p* = 0.041) and achievement of confirmed virological success at least once within the first two years of ART (OR = 3.0 (1.3–6.9), *p* = 0.010). There was a non-significant association between VS at M60 and controlled viral load (VL < 400 copies/mL) at two years of ART initiation (OR = 2.2 (0.9–4.8), *p* = 0.061), being followed at “Centre Hospitalier d’Essos” versus “Centre Mère et Enfant de Yaoundé” and “Hôpital Laquintinie de Douala” (OR = 2.8 (1.0–7.6), *p* = 0.056) and absence of missed doses during the last three days before the M60 visit (OR = 2.8 (0.9–8.7), *p* = 0.076).

**Table 3:** Factors associated with virological success (Viral load < 400 copies/mL versus > 400 copies/mL or not measured) in HIV-infected children at 5 years after early antiretroviral treatment initiation (ANRS-Pediacam Study, 2008–2013, Cameroon)

Baseline characteristics at 2 years +/− 3 months after ART initiation	Univariate analysis VL < 400 cp/mL at 5 years +/− 3 months after ART initiation					Multivariate analysis (logistic regression)		
	N	n	%	Crude OR (95%CI)	p	Adjusted OR (95%CI)	p	N2
								142
**Gender**								
Male	65	39	60.0	1	0.041		0.122	
Female	79	60	76.0	2.1 (1.0–4.4)		1.9 (0.9–4.2)		
**Site of ART delivery**								
CME/FCB	74	47	63.5	1	0.056	1	0.102	
HLD	30	19	63.3	0.8 (0.3–18)		0.7 (0.3–1.8)		
CHE	40	33	82.5	2.8 (1.0–7.6)		2.4 (0.8–7.2)		
**Age**								
< 28 months (treatment initiated before age of 4 months)	72	50	69.4	1	0.855			
≥ 28 months (treatment initiated from age of 4 months)	72	49	68.1	0.9 (0.5–1.9)				
**Calendar period**								
2010–2011	91	63	69.2	1	0.923	1	0.206	
2012–2013	53	36	67.9	1.0 (0.5–2.0)		1.8 (0.7–4.4)		
**Virological response within the first two years of ART**								
Absence of confirmed virological success	29	15	51.7	1	0.010	1	0.033	
Achievement of confirmed virological success at least once (= 2 VL < 1000 copies/mL for two consecutive measurements)	115	84	73.0	3.0 (1.3–6.9)		2.7 (1.1–6.8)		
**Virological status**								
VL ≥ 400 copies/mL or not measured	54	29	53.7	1	0.061			
VL < 400 copies/mL	90	70	77.7	2.2 (0.9–4.8)				
**Children living with**^(1)^								
Mother or father	96	77	80.2					
Other relatives	23	23	100.0					
**Availability of refrigerator in the household**								
No	40	34	85.0	1	0.939			
Yes	44	36	81.8	1.0 (0.4–2.6)				
**Access to electricity at home**^(1)^								
No	5	3	60.0					
Yes	79	67	84.8					
**Access to water at home**^(1)^								
No	52	42	80.9	1	0.876			
Yes	32	28	87.5	1.0 (0.4–3.0)				
**History of hospitalization, diarrhea, infectious disease, or convulsions within the last 3 months before the M60 visit**								
No	75	59	78.7	1	0.975			
Yes	45	37	82.2	1.0 (0.5–2.3)				
**CD4 percentage**^(2)^								
< 25%	30	29	96.2	1	0.221			
≥ 25%	89	70	78.7	0.5 (0.2–1.5)				
**ART regimen**								
Lopinavir-based	106	77	72.6	1	0.117	1	0.155	
Nevirapine-based	38	22	57.9	0.5 (0.2–1.2)		0.5 (0.2–1.3)		
**Number of missed doses within the last 3 days before M60 visit**								
≥ 1 missed dose	95	77	81.1	1	0.076	1	0.075	
No missed dose	30	22	73.3	2.8 (0.9–8.7)		2.7 (0.9–10.4)		

*VL* viral load; *ART* antiretroviral therapy: *OR* odds ratio; 95%CI: 95% confidence interval; *N* total number of subjects at 5 years after treatment initiation; n: number of subjects with virological success at 5 years after treatment initiation; p: statistical test; *N2* total number of subjects in final multivariate model; VL: viral load; ^(1)^: Variable excluded from the univariate and multivariate analyses because of unbalanced sizes of categories; ^(2)^: Variables excluded from the multivariate analysis because of collinearity with virological status at 2 years after treatment initiation

In multivariate analysis (Table 3), achievement of confirmed virological success at least once within the first two years of ART was associated with VS at five years of treatment (adjusted OR = 2.7 (1.1–6.8); *p* = 0.033). The trend between VS and absence of missed doses during the last three days before the M60 visit remained (adjusted OR = 2.7 (0.9–10.4), *p* = 0.075).

## Discussion

The proportion of VS among children still alive and followed-up at five years of treatment, initiated before the age of 12 months, was 66.8% (60.1–73.5), comparable to the results reported for Italian children who initiated ART early [43], and adult populations [44]. This result contrasts with a lower proportion of VS reported in a study conducted on children in Ivory Coast who initiated ART at a higher median age (36 months versus 4 months in our study) [45].

Among children alive at two years of treatment, 23 (15.4%) did not have viral load measurements at five years whilst they were still alive. Active telephone recalls of non-returning families made it possible to hear regularly from these children [46]. The low mortality beyond the first two years of antiretroviral treatment (3.3% (95%CI: 0.4–6.2)) is coherent with the results of other studies conducted in children and adults. Most deaths occurred during the first year of ART [9, 28, 44, 47–49]. The high mortality observed in the cohort before age 2 years of ART may reflect the rapid progression of the disease in infants enrolled in medical care after severe immunodeficiency had set in [42].

The probability of maintaining VS between two and five years of ART, estimated at 64.0% (54.0–74.0), highlights the difficulty of parents to administer drugs daily to their children [42]. The only factor independently associated with a controlled viral load (< 400 copies/mL) at five years of treatment was the existence of VS at two years of treatment, irrespective of living conditions. The association with VS at two years of ART may be related to factors that favor long-term family adherence to ART or favorable immunological or virological factors in these children [50].

One of the strengths of our study was the use of data from a prospective cohort of children, with scheduled determinations of viral load at six-month intervals, making it possible to use survival models.

Some limitations of this study included the use of current missed doses self-reported by the child caregiver. It may not be a good stool for detecting virological success at 5 years of ART. It does not reflect the longitudinal adherence evolvement. It has been demonstrated previously that the negative predictive value of current missed dose in detecting virological success [36] was as satisfactory as cumulative missed doses (around 75%). In fact, virological response seems to be mostly influenced by adherence status within the last three months, especially when there is no drug resistance. The non-significant association between the absence of missed doses during the last three days before the M60 visit and virological success at this deadline would become significant if the study population size was larger. This hypothesis would confirm the difficulty of parents to administer daily medications to their children, given the difficulty in this study to maintain virologic suppression in children who had initiated ART early during infancy. We did not obtain exhaustive genotyping results for all the children in this study. The HIV-resistance patterns were similar, irrespective of virological status at five years of treatment, among the 25 children who were tested for genotyping. It is not possible to draw conclusions on the effect of HIV resistance mutations on the virological response at five years of ART early initiated during infancy because the study was not sufficiently powered to address this issue.

## Conclusions

This study reported difficulty in maintaining virologic success in sub-Saharan HIV-infected children between two and five years of early initiated ART. It also highlighted the importance of initial viral suppression for achieving and maintaining virologic success in the long-term. Further studies should focus on identifying strategies that would enhance better retention in care and improved adherence to treatment within the first two years of ART early initiated in Sub-Saharan HIV-infected children.

## Data Availability

The datasets used and/or analyzed during the current study are available from the corresponding author on reasonable request.

## Abbreviations

ART: Antiretroviral therapy
HIV: Human Immunodeficiency Virus
ANRS: French National Agency for Research on AIDS and Viral Hepatitis
VS: Virologic success
M24: 24 months after ART initiation
M30: 30 months after ART initiation
M36: 36 months after ART initiation
M42: 42 months after ART initiation
M48: 48 months after ART initiation
M54: 54 months after ART initiation
M60: 60 months after ART initiation
95%CI: 95% confidence interval
